# Prognostic Value of Doppler Ultrasound in Triplets Conceived by In Vitro Fertilization: A Case Report and Review of the Literature

**DOI:** 10.1177/2324709619864131

**Published:** 2019-07-17

**Authors:** Rita Zafra, Lauren Conway, Nadia Solomon

**Affiliations:** 1 Icahn School of Medicine at Mount Sinai, Queens Hospital Center, Jamaica, NY, USA; 2 St. George’s University, True Blue, Grenada

**Keywords:** Doppler, high-risk pregnancy, maternal-fetal medicine, multiple pregnancy, ultrasound

## Abstract

Umbilical artery Doppler ultrasound—which informs on maternal-fetal-placental blood flow—may be the most valuable surveillance tool in triplet pregnancies, crucial to diagnose early fetal growth restriction. To illustrate the prognostic value of Doppler ultrasound and launch a discussion of its role in multiple gestational, high-risk pregnancy, we present the case of a 42-year-old woman with trichorionic triamniotic triplet pregnancy conceived by in vitro fertilization, who showed early signs of poor outcome for one of the triplets via umbilical artery Doppler by the 23rd week and middle cerebral artery Doppler by the 29th week of gestation.

## Introduction

Umbilical artery (UA) Doppler ultrasound (US)—which informs on maternal-fetal-placental blood flow—may be the most valuable surveillance tool in triplet pregnancies, as it plays a crucial role in the diagnosing of early fetal growth restriction (FGR).^1,2^ In the following case report, we present a 42-year-old G1P0 woman with a triplet pregnancy (TP) complicated by a number of factors. This is used to launch a discussion of the prognostic value of Doppler US, especially in multiple gestation pregnancies, with a review of the literature to comprehensively analyze the high-risk nature of this case.

## Case Report

A 42-year-old G1P0 woman from Sudan presented for prenatal care with trichorionic triamniotic (TT) TP, conceived via in vitro fertilization (IVF) with three 5-day blastocysts in Nairobi, Kenya. She had previously undergone uterine myomectomy and unsuccessful attempts at IVF. US records from 7 weeks 6 days (7w6d) gestation confirmed 3 intrauterine pregnancies, with additional confirmation by crown-rump length. Her uterus was enlarged by multiple fibroids. She started prenatal care at 22w2d gestation with a 33-cm uterus.

**Figure 1.**
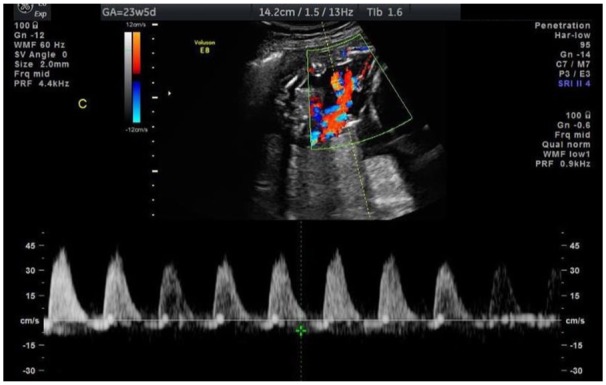
Umbilical artery Doppler ultrasound at 23w5d revealing reversed diastolic flow in Fetus C.

At 23w5d, US confirmed TT triplets with separating membranes. Fetus A was 24w6d with estimated fetal weight (EFW) of 696 g (58th percentile); Fetus B was 23w4d with EFW of 603 g (40th percentile); and Fetus C was 19w2d with EFW of 209 g (<10th percentile). All fetuses demonstrated normal limited anatomy and movements. UA Doppler, however, revealed reversed flow in Fetus C (Figure 1). US also showed a posterior leiomyoma in the lower uterine segment measuring 13.2 × 9.2 cm, near Fetus C. UA Doppler findings were confirmed at 24w5d.

**Figure 2.**
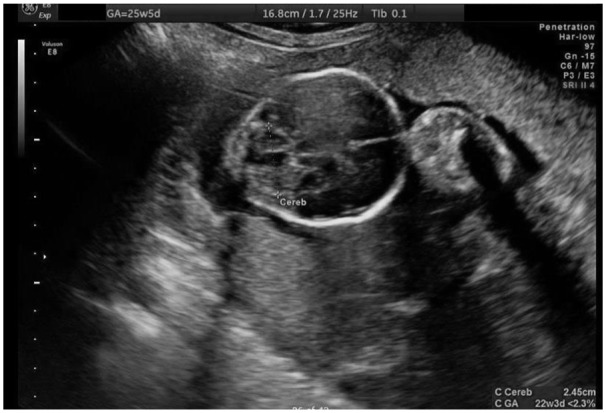
Ultrasound at 25w5d demonstrating posterior fossa with cyst/absent cerebellar vermis in Fetus C.

At repeat US 2 weeks later, Fetus C demonstrated abnormal heart, cardiomegaly, posterior fossa with cyst/absent cerebellar vermis (Figure 2), displaced urinary bladder, 2 vessel cord, subcutaneous edema, and developing hydrops. UA Doppler revealed absent end diastolic flow (AEDF) without reversal. The patient was referred for fetal echocardiogram (echo) and follow-up US. Amniocentesis and genetic testing were declined.

Initial fetal echo, delayed due to insurance issues, took place at 28w5d. Echoes were normal for Fetuses A and B, whereas Fetus C—small, with discordant biparietal diameter (24.2w) and fetal length (19.4w)—demonstrated levocardia and situs solitus, UA AEDF, and elevated diastolic velocity in the middle cerebral artery (MCA) consistent with brain-sparing autoregulation. Diffuse biventricular hypertrophy, prominent right ventricular trabeculations (suggesting myocardial non-compaction cardiomyopathy), hyperdynamic systolic function, and a small pericardial effusion were noted. Biphasic filling pattern was normal, without atrioventricular regurgitation. Fetal heart rate was normal with 1:1 atrioventricular conduction.

Repeat US at 33w5d revealed growth of all triplets, with abnormalities consistent with prior examinations. The fetuses were monitored weekly from then onward.

Uncomplicated cesarean section took place at 34w6d. Baby A, male, was delivered breech, 2425 g, APGAR 9 and 9. Baby B, male, was delivered cephalic, 1960 g, APGAR 9 and 9. Baby C, sex undetermined, was delivered breech, 748 g, APGAR 7 and 9. Postoperatively, the mother demonstrated stable vitals and was discharged with Baby A. Babies B and C remained in the neonatal intensive care unit. Baby B was eventually discharged, but Baby C demonstrated numerous congenital abnormalities, including a prominent forehead, 2 vessel cord, ambiguous genitalia with small phallus, hypospadias, and bifid empty fold below the phallus.

At day 9, Baby C deteriorated, requiring intubation, high-frequency oscillatory ventilation, and antibiotics. X-ray revealed free intraperitoneal air. Drop in heart rate and metabolic acidosis prompted full cardiopulmonary resuscitation, and hematocrit of 29 necessitated transfusion of packed red blood cells. Baby C, however, did not respond. At autopsy, cause of death was identified as necrotizing enterocolitis due to prematurity.

## Discussion

Numerous features contributed to the high-risk nature of this pregnancy. Multiple gestation pregnancies carry increased risks of FGR and growth discordance, surveillance and interventions, preterm birth, and neonatal mortality and morbidity.^3^ A study of 10 000 women undergoing IVF up to 6 cycles found a cumulative live birth rate for triplets of only 1.3%^4^; and a study on chorionicity found that TT triplets carry a neonatal death rate of 1.3% (significantly lower than MT [monochorionic triamniotic] at 2.7%, but higher than DT [dichorionic triamniotic] at 0.5%).^5^ Presence of an anomalous triplet (ie, with congenital defects) is also associated with increased perinatal mortality of that triplet and cotriplets.^6^ In cases where there are fetal structural abnormalities in combination with apparent FGR, current American College of Obstetricians and Gynecologists guidelines recommend genetic counseling and prenatal diagnostic testing as the structural abnormalities may be multifactorial or due to a genetic abnormality.^7^ The earlier the onset of FGR, the higher the likelihood of a genetic abnormality as the cause, as first trimester fetuses with an aneuploidy, especially trisomy 13 and 18, have been shown to be growth restricted.^8^ Although the mother in this case refused amniocentesis and genetic testing, addition of this testing may have elucidated an additional cause of FGR besides the presence of a structural abnormality with reduced maternal-fetal-placental blood flow, since Fetus C presented with FGR at 19w2d (possibly earlier, but onset of prenatal care was delayed).

Ultrasound is currently the preferred modality for diagnosing growth anomalies, although its use to detect growth anomalies in triplets is currently suboptimal.^3^ Sklar et al suggested modifying the diagnostic criteria by raising the threshold for intrauterine growth restriction in triplets from the 10th to the 15th percentile, lowering the threshold for growth discordance from 25% discordance to 20%, and revising the definition of intrauterine growth restriction to include specifiers for a significant decrease in growth velocity. These factors could ultimately increase the sensitivity of US in predicting outcomes and depend on continued usage of Hadlock’s formula.^3^ Although there are newer equations used to measure EFW, Hadlock’s formula, when utilizing the Bland-Altman test to confirm, still performs well and should still be used for clinical decision-making in multi-gestational pregnancies.^9^

Fetal growth restriction, as observed in Fetus C, is the second leading cause of prenatal morbidity and mortality after prematurity.^2^ Studies have shown that a weight discordance of >20% results in an increase in perinatal morbidity and mortality.^9^ Prenatal US, when utilized with Hadlock’s formula—which takes into account head circumference, abdominal circumference (AC), and femur length^10^—is the most reliable screening tool for growth anomalies in TP, and is as reliable in triplet as in twin or singleton pregnancies,^3,9^ with a recent study reporting specificity, positive predictive value, and negative predictive value of 100% for US to predict ≥1 fetus with FGR in triplets, and 80% sensitivity, 94.1% specificity, 66.7% positive predictive value, and 97.0% negative predictive value of US for detecting >25% EFW discordance between triplets.^3^ Measurements of EFW discordance ≥25%, specifically measurements ≥29% in triplets, are understood to be predictive of increased mortality in multigestational pregnancies.^3^ Detection of fetal growth rate abnormalities via first or second trimester US screening prompt Doppler studies, and these studies in conjunction with maternal characteristics have been shown to detect early-onset growth restriction in 90% of cases.^11^ Doppler studies in these instances reduce stillborn births without increasing neonatal mortality.^11^

In FGR, Doppler US informs on maternal-fetal-placental blood flow.^1,2^ Arterial Doppler waveforms reflect vascular resistance and provide insight on the distribution of cardiac output.^2^ Venous Doppler waveforms assess afterload, compliance, and contractility that mirrors cardiac function.^2^ Placental insufficiency shown by impaired gas exchange at the level of the placenta resulting in hypoxia and acidosis defines true FGR, whereas small for gestational age is caused by anomalies unrelated to the placenta such as large leiomyoma,^12^ aneuploidy, or infection.^11^ Both result in neurodevelopmental, cardiovascular, and endocrine abnormalities,^11^ all of which were present in Fetus C. It is important to note that although a large leiomyoma (>5 cm) was present during pregnancy, myomectomy has not been proven to improve obstetric or delivery outcomes during pregnancy, although it has been shown to improve pregnancy rates and decrease miscarriage rates when performed prior to pregnancy.^12^ Current guidelines recommend counseling patients on poor obstetric outcomes with elective myomectomy on a case-by-case basis due to the increased risk of hemorrhagic and traumatic complications.^12^

Umbilical artery Doppler may be the most valuable surveillance tool in TP,^6^ and is crucial to diagnose early FGR because it provides information on the villous branching in the fetal side of the placenta.^2^ In healthy pregnancies, UA Doppler waveforms follow a low-resistance “saw-tooth” pattern with forward flow throughout diastole and Doppler indices that decline with gestational age due to increasing numbers of villous branching.^2^ This results in a decrease in resistance on the fetal side of the placenta, where early in pregnancy resistance was high due to incomplete invasion of villous branching.^1,2^ The umbilical vein Doppler reveals a monophasic pattern and velocity is maintained throughout the pregnancy.^1,2^

In studies associated with TP, adverse pregnancy outcomes have been previously associated with abnormal UA waveforms characterized by absent end-diastolic velocities.^13^ Abnormal UA waveforms are a criterion for FGR. These waveforms show absent or reversed end-diastolic flow, or an increased pulsatility index (PI) >95th percentile, which is a semiquantitative, low-error measurement with a narrow reference limit and the preferred index.^2,11^ Increased UA resistance can present with a decrease or reversal in UA end-diastolic flow.^2^ Deficient UA diastolic flow and pulsatile umbilical vein flow suggest fetal distress, and reversed diastolic flow often signifies fetal cardiac decomposition^1^ resulting from severe hypoxia and increased placental resistance.^2^ In the second trimester, increased UA resistance due to diminished trophoblastic villous vascular area has been associated with right-sided heart defects; and brain-sparing and/or increased UA resistance may be seen with transposition of the great vessels and other heart defects (ie, hypoplastic left heart syndrome, severe aortic stenosis).^2^ Studies in 2 year olds who were born preterm have demonstrated a correlation between cognitive impairment, compromised motor development, and absent or reversed UA-AEDF.^14^

Early-onset FGR (gestational age <32 weeks, EFW/AC <3rd centile and/or UA-AEDF, or EFW/AC <10th centile with uterine artery PI >95th centile and/or UA-PI >95th centile)^11^ has been linked to inappropriate trophoblastic spiral artery invasion and impaired placentation.^2^ By the second trimester, diminished trophoblastic villous vascular area results in increased UA resistance, especially when FGR is early-onset and severe.^2^ Studies on Doppler US and FGR associate abnormal UA Doppler with adverse perinatal outcome, regardless of EFW or AC,^15^ with severe abnormal UA waveforms associated with a 10-fold increase in perinatal mortality, and placental ischemia or velamentous cord insertion.^6^ Late-onset FGR (gestational age >32 weeks, EFW/AC <third percentile, or 2 out of 3 of the following: EFW/AC <10th centile, EFW/AC crossing centiles >2 quartiles on growth centiles, and cerebroplacental ratio (CPR) <5th or UA-PI >95th centile)^11^ is more common than early-onset FGR, easier to manage and more difficult to diagnose. In these cases, the trophoblastic villous vascular area fails to mature although the surface area is unaffected resulting in impaired gas and nutrient exchange causing a hypoxic state.^11^ MCA-PI in conjunction with UA-PI is a useful tool in diagnosing late-onset FGR because it can show decreased resistance in the MCA.^11^

In either case, early-onset or late-onset, current American College of Obstetricians and Gynecologists guidelines recommend that delivery should take place: (1) between 32w0d and 37w6d in fetuses with FGR and concerning fetal findings such as absent (34w0d) or reversed end-diastolic flow (32w0d) seen on UA Doppler for singleton cases, and (2) 38w-38w6d for dichorionic-diamniotic twins. For triplet cases, time of delivery is decided on an individual basis with delivery taking place between 32w0d and 37w6d.^7^ For this case, a cesarean section was performed at 34w6d.

In a normal pregnancy, MCA Doppler should reflect high-resistance flow,^2^ with resistance measured with PI (the difference between peak systolic and end-diastolic velocities).^2^ In FGR, there is low-resistance flow in the MCA due to brain-sparing, a phenomenon where fetuses will divert oxygen and nutrient-rich blood from organs deemed nonessential to essential organs such as the brain in hypoxic states.^2^ This phenomenon may be demonstrated by low PI, which reflects low-resistance MCA systolic flow and a rise in MCA diastolic flow, with a decline in UA diastolic flow.^1^ In the context of morbidity and mortality, a study of 2518 pregnancies in which US assessment was performed at 34^+0^-35^+6^ weeks found that UA PI multiples of the median were significantly higher, and MCA PI and CPR (ratio of MCA PI to UA PI) multiples of the median were significantly lower in neonates requiring neonatal unit admission.^16^ Animal models have consistently demonstrated that CPR better represents hypoxia than MCA-PI or UA-PI alone.^17^ In this case, UA Doppler suggested poor prognosis for Fetus C as early as 23 weeks’ gestation, and MCA Doppler elaborated on these findings at first fetal echo in the 29th week, emphasizing the prognostic value of UA and MCA Doppler in these triplets and suggesting Doppler US can predict outcomes relatively early in pregnancy.
